# Incorporating breath holding and image guidance in the adjuvant gastric cancer radiotherapy: a dosimetric study

**DOI:** 10.1186/1748-717X-7-98

**Published:** 2012-06-20

**Authors:** Weigang Hu, Jinsong Ye, Jiazhou Wang, Qing Xu, Zhen Zhang

**Affiliations:** 1 Department of Radiation Oncology, Fudan University Shanghai Cancer Center, Department of Oncology, Shanghai Medical College, Fudan University, Shanghai, China, 200032; 2 Department of Radiation Oncology, Swedish Caner Institute, Seattle, WA, USA

**Keywords:** Gastric cancer, Intensity-modulated radiotherapy, Breath holding, Image-guided radiotherapy, Dose convolution

## Abstract

**Background:**

The respiratory related target motion and setup error will lead to a large margin in the gastric radiotherapy. The purpose of this study is to investigate the dosimetric benefit and the possibility of incorporating the breath-hold (BH) technique with online image-guided radiotherapy in the adjuvant gastric cancer radiotherapy.

**Methods:**

Setup errors and target motions of 22 post-operative gastric cancer patients with surgical clips were analyzed. Clips movement was recorded using the digital fluoroscopics and the probability distribution functions (pdf) of the target motions were created for both the free breathing (FB) and BH treatment. For dosimetric comparisons, two intensity-modulated radiotherapy (IMRT) treatment plans, i.e. the free breathing treatment plan (IMRT_FB_) and the image-guided BH treatment plan (IMRT_IGBH_) using the same beam parameters were performed among 6 randomly selected patients. Different margins for FB and BH plans were derived. The plan dose map was convoluted with various pdfs of the setup errors and the target motions. Target coverage and dose to organs at risk were compared and the dose-escalation probability was assessed.

**Results:**

The mean setup errors were 1.2 mm in the superior-inferior (SI), 0.0 mm in the left-right (LR), and 1.4 mm in the anterior-posterior (AP) directions. The mean target motion for the free breathing (vs. BH) was 11.1 mm (vs. 2.2 mm), 1.9 mm (vs. 1.1 mm), and 5.5 mm (vs. 1.7 mm) in the SI, LR, and AP direction, respectively. The target coverage was comparable for all the original plans. IMRT_IGBH_ showed lower dose to the liver compared with IMRT_FB_ (p = 0.01) but no significant difference in the kidneys. Convolved IMRT_IGBH_ showed better sparing in kidneys (p < 0.01) and similar in liver (p = 0.08).

**Conclusions:**

Combining BH technique with online image guided IMRT can minimize the organ motion and improve the setup accuracy. The dosimetric comparison showed the dose could be escalated to 54 Gy without increasing the critical organs toxicities, although further clinical data is needed.

## Background

The gastric carcinoma is one of the leading causes of cancer death in China. Traditionally, radiation therapy has played a limited role in the management of gastric tumors [1]. The Southwest Oncology Group study INT0116 showed that the adjuvant chemotherapy with concurrent radiation had significant benefit in overall survival compared to surgery alone, however, the acute toxicity was high mainly due to the AP/PA field arrangement used in the radiation treatment [2]. Compared with the technique used in the INT-0116 study, the 3D conformal radiotherapy (3DCRT) and intensity modulated radiation therapy (IMRT) can largely reduce the dose to the surrounding dose-limiting structures such as liver and kidneys [3,4]. However, even with these advanced treatment techniques, a large margin is still needed to account for the setup uncertainties and the target motions. This limits the dose that can be safely delivered to the target and potential dose escalation in gastric cancer radiation.

Patient daily setup accuracy can be improved with the daily image guidance correction [5]. However, the respiratory related target motions are of major concerns for abdominal tumors. Data from 4D CT and fluoroscopy have shown that the organs and tumors in the abdominal region can move 10–27 mm in a normal breathing cycle [6]. Various methods have been proposed to reduce the respiratory induced target motion, such as the active or passive breath-hold techniques, abdominal compression, respiratory gating and tracking [7-9]. The active breathing control (ABC) is one of breath-hold (BH) methods, which can reduce the target motion by temporarily suspending the patient’s breath during the treatment delivery.

The static dose can be convoluted with the probability distribution functions (pdf) of the motion for incorporating the motion to obtain a really dose distribution. Such method has been used to investigate the motion effect in many sites such as liver and pancreas [10,11]. But, the impact of target motion on the IMRT and the dosimetric benefit of combining breath-hold technique with the image guidance in the gastric cancer radiotherapy have not been reported. The purposes of this study are to 1) quantify the target motion in the gastric cancer treatment with or without the breath-hold technique, 2) evaluate the effect of the setup uncertainties and target motion on the planned dose, 3) assess the possibility of dose escalation with image guided breath-hold IMRT.

## Methods

### Patient selection and CT imaging

Twenty-two patients diagnosed with T3-4 and/or N+, staging II-IV gastric cancer and received post-operative radiation therapy between July and December 2008 were enrolled in the study. The study was reviewed and approved by the institutional ethics committee. Four to six surgical clips used as target-motion surrogates were implanted near the tumor bed during the surgery prior to the radiotherapy. Customized vacuum lock bags (Med-Tec Corporation, USA) were used for patient immobilization. Each patient went through an ABC (Elekta Oncology Systems, Crawley, UK) training session with an experienced therapist during the simulation.

The planning CT images were acquired in 5-mm slice thickness on a large bore CT scanner (Philips Medical Madison, WI) with ABC. The volume threshold for breath hold was set to 75% of the deep inspiration. A radiation oncologist delineated the clinical target volumes (CTVs) and critical structures such as liver, kidneys and spinal cord. The CTV included the tumor bed and the regions of lymphatic drainage.

### Setup error and target motion

The setup error and target motion were evaluated by weekly digital fluoroscopic imaging throughout the treatment course. Patient setup was mimicked on a simulator (Ximatron, Varian Medical) weekly by two therapists who actually positioned the patient on the treatment machine everyday. For the free breathing (FB) treatment, the patient was instructed to breathe normally. Several series of the anterior-posterior and lateral orthogonal fluoroscopic images were acquired to cover several breathing cycles after the patient had established a stable and reproducible breathing pattern. For the breath-hold (BH) treatment, patients were instructed to hold their breath with the aid of ABC and 3–4 consecutive series of fluoroscopic imaging were acquired for each patient with two-minute intervals during each simulation session.

The fluoroscopic images were registered to the digitally reconstructed radiography (DRR) images generated by the treatment planning system (TPS, Philips Medical Systems, Pinnacle v7.6c, Milpitas, CA) based on the vertebral column. As the patients were initially set up to the skin marks, the setup errors were derived from the registration shifts ignoring the rotation errors. Additionally, the clips on each frame of the images were traced and recorded to calculate the trajectory of the target motion under FB mode, or the residual motion for the BH mode, assuming the target-motion related clip deformation was negligible.

The centroid location of the clips representing the target motion was calculated on each frame. The fluoroscopic images were captured at a rate of 3 frames per second.

### Probability distribution function

The displacement of the centroid was plotted against the time (the amplitude of the target motion versus time), which could be converted to the probability distribution function (pdf) of the target motion (probability versus position). A pdf was derived from the weekly orthogonal fluoroscopic images and used to calculate the accumulative dose that the patient received for that week.

### Dose comparison and escalation

The setup error can be regarded as independent of the target motion during the treatment delivery, thus the quadratic model was used to estimate the CTV to planning target volume (PTV) margin for treatment planning. This total margin consists of the setup margin (SM) and the internal margin (IM), i.e. $\text{M}=\sqrt{{\text{SM}}^{2}+{\text{IM}}^{2}}$. Different PTVs (PTV_FB_ and PTV_IGBH_) were generated from the same CTV for each patient using different margins: treatment delivery in the FB condition without image guidance (“FB” scenario) and treatment delivery with online image-guided BH condition (“IGBH” scenario). Note that the PTV margins were calculated based on the setup errors and target motion data collected from twenty-two patients. For each patient, two plans (IMRT_FB_ for PTV_FB_ and IMRT_IGBH_ for PTV_IGBH_) were generated with 7–9 coplanar fields and maximum 35–45 segments, and the same dose prescription, beam orientation and objective functions were used in the optimization. The prescription was 45 Gy delivered in 25 fractions. The final treatment plans should meet the following criteria: at least 95% of PTV (PTV_FB_ and PTV_IGBH_) received 45 Gy and ≥99% of PTV received 42.75 Gy; less than 30% of the liver received 26 Gy; no more than 33% of any kidney received ≥18 Gy; and the spinal cord dose ≤45 Gy. The final dose matrices were exported to program ImageJ (NIH, http://rsbweb.nih.gov/ij/) for 3D convolution calculations.

The details were listed as following:

(1) Extract the 3D dose matrix for five fractions of treatment from the original treatment plans (IMRT_FB_ and IMRT_IGBH_) in the Pinnacle Planning System. The 3D dose matrix was recorded as 32-bit real numbers in big endian format.

(2) Read this 3D dose matrix for five fractions in Image J program. The 3D dose matrix was expressed as a stack of 2D transverse dose planes in Image J;

(3) Import the weekly pdfs in the Image J. The weekly pdf was the probabilities of the target at each position along three directions, AP, LR and SI, for five fractions of treatment;

(4) Convolute the 3D dose matrix with the weekly pdfs using the “Convolution 3D” function in Image J program. For the free breathing scenario, the static weekly dose matrix from the original static IMRT_FB_ plan was convoluted with the pdfs for the setup errors (pdf_SetupFB_) and for the free breathing target motion (pdf_FB_) derived from the weekly fluoroscopic imaging; and for the image-guided breath hold scenario, the static weekly dose matrix of the IMRT_IGBH_ plan was convoluted with the weekly pdf of the residual target motion for multiple breath hold (pdf_BH_) and the residual setup errors after image guidance (pdf_SetupBH_).

(5) Pinnacle scripts were written to import the motion incorporated dose distribution (result of last step) for analysis and evaluation.

**Figure 1:**
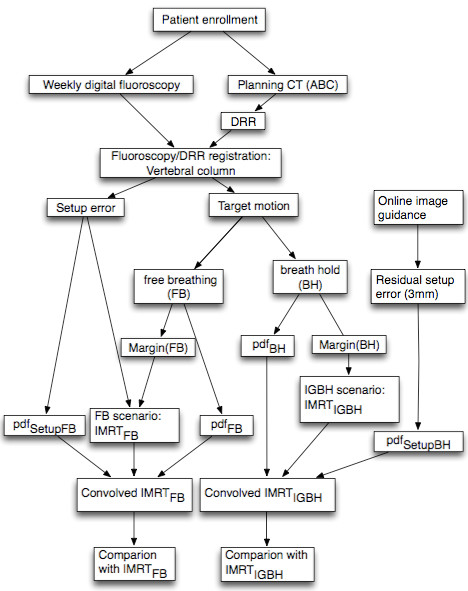
The diaphragm of this study.

The target coverage and dose to organs at risk for both scenarios were compared using the two-sided Wilcoxon matched-pair signed-rank test. It was considered statistically significant if p ≤ 0.05. Additionally, the prescription dose was increased from 45 Gy to 54 Gy in the IMRT_IGBH_ plans to investigate the probability of dose escalation. Figure 1 shows the scheme of this study.

## Results

### Setup uncertainties

A total of 220 pairs of orthogonal fluoroscopic images were acquired for the 22 patients. The mean (simple average) and stand deviation of the setup variations along the superior-inferior (SI), left-right (LR) and anterior-posterior (AP) directions are 1.2 ± 3.4 mm, 0.0 ± 1.8 mm, and −1.4 ± 3.1 mm, respectively. The margin for setup error (M_Setup_) is 8.3 mm in the SI direction, 3.3 mm in the LR direction, and 6.4 mm in the AP direction calculated with the margin recipe proposed by Van Herk, et al. [12].

### Target motion

#### Free breathing scenario

**Figure 2:**
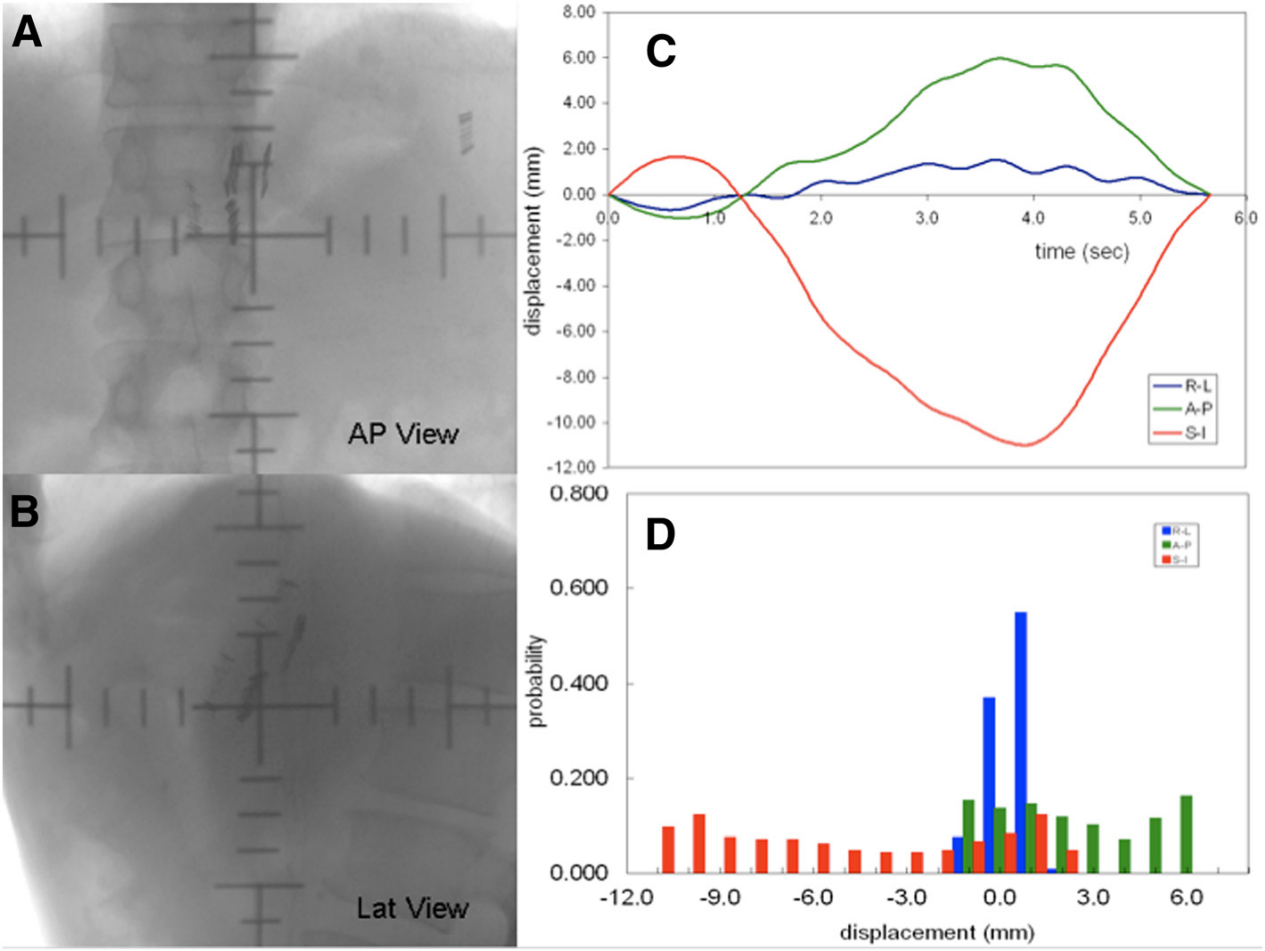
**The clips motion trajectory after their positions on each frame were projected on to the reference frame (A: AP direction; B: LR direction) and the displacement of the centroid clips versus time in SI, LR, and AP directions (C).D: Weekly Probability Distribution Functions In The Si, Lr And Ap Direction For The Free Breathing Treatment.**

Figure 2.A and B depict the motion trajectory of the clips on the AP and LR views in the free breathing condition for one patient (Patient5). In this figure 20 frames of the fluoroscopic images were projected to a reference frame (the end of inspiration image). The target motion was represented by the motion of the centroid of clips, assuming that the relative positions between any two clips did not change significantly. The displacement of the centroid versus the time for one breathing cycle along the AP, LR and SI directions for this patient is illustrated in Figure 2.C. The mean values of the motion excursion are 11.1, 1.9, and 5.5 mm in the SI, LR, and AP direction, respectively. The margin to compensate for the target motion in free breathing, calculated as M_FB_ = Mean +3SD, is 20.4 mm in the SI direction, 4.9 mm in the LR direction, and 13.0 mm in the AP direction to ensure that 99.7% of the times the target falls within this margin.

#### Breath-hold scenario

The maximum residual target motion in the SI, LR and AP directions are 10.1 mm, 3.5 mm and 8.1 mm, respectively. The mean values of the motion excursion are 3.7, 1.6, and 2.8 mm in the SI, LR, and AP direction, respectively. The frequency of occurrence for the motion less than 6 mm is 86.1% in SI, 91.4% in AP and 100% in the LR direction. Based on the formula M_BH_ = Mean +3SD, a margin of 9.7 mm in the SI direction, 4.0 mm in the LR direction, and 7.9 mm in the AP direction is needed to cover the target for multiple breath-hold treatment.

The detail target motion data in both free breathing and breath-hold scenarios are listed in the Table1.

**Table 1:** The range, mean and standard deviation (SD) of the target motion represented by the movement of the centroid of the clips in free breathing and breath-hold conditions

		**SI(mm)**	**LR (mm)**	**AP (mm)**
Free breathing	Max	20.0	5.8	12.5
	Min	2.8	0.8	2.2
	Mean	11.1	1.9	5.5
	SD	3.1	1.0	2.5
	Margin	20.4	4.9	13.0
Breath hold	Max	10.1	3.5	8.1
	Min	1.0	0.3	0.8
	Mean	3.7	1.6	2.8
	SD	2.0	0.8	1.7
	Margin	9.7	5.0	7.9

The margin for target motion was calculated using three times of the SD. All data are calculated from the entire group of the patients.

### Probability density functions

The probability distribution functions of the target motion in free breathing (pdf_FB_) and breath-hold (pdf_BH_) treatment describe the probability of the target at each position in the entire motion excursion. Figure 2.D shows the corresponding pdf_FB_ for one of the patients (Patient5). For the patient daily setup variations, both pdf_SetupFB_ and pdf_SetupBH_ were generated by using the Gaussian distributions.

### Dose comparison for the free breathing and breath-hold treatments

The PTV_FB_ margin for free breathing treatment planning was calculated to be 22.0, 5.9 and 14.5 mm in the SI, LR and AP direction, respectively. For the image-guided breath-hold treatment, a 3.0-mm setup margin was used to account for the residual error introduced by the online correction strategy. The internal margin (M_BH_) was used to cover the residual target motion for multiple breath-hold periods. The overall margins for PTV_IGBH_ were 10.2 mm in the SI direction, 5.0 mm in the LR direction and 8.5 mm in the AP direction. The comparison was performed on 6 randomly selected patients. Table2 lists the comparison of the target coverage and the dose to organs at risks for the original static plans and motion convoluted plans between free breathing and breath-hold treatment.

**Table 2:** Comparison of the delivered dose to CTV and organs at risk for free breathing and breath-hold treatment techniques: convolution with the probability density functions

		**CTV**		**Liver**	**L kidney**	**R kidney**
		V_45_	V_42.75_	V_30_	V_15_	V_15_
		Mean ± SD (%)	Mean ± SD (%)	Mean ± SD (%)	Mean ± SD (%)	Mean ± SD (%)
Static plan	IMRT_FB_	99.7 ± 0.5	100.0 ± 0.1	N1*	N1*	N1*
	IMRT_IGBH_	99.7 ± 0.6	100.0 ± 0.0	−5.6 ± 4.7	−6.0 ± 8.8	−5.4 ± 7.9
	p value	0.96	0.76	0.01	0.09	0.09
Convoluted plan	IMRT_FB_	99.7 ± 0.3	100.0 ± 0.1	N2*	N2*	N2*
	IMRT_IGBH_	99.8 ± 0.4	100.0 ± 0.0	−3.8 ± 5.4	−14.8 ± 9.9	−11.0 ± 7.7
	p value	0.78	0.33	0.08	<0.01	<0.01

N1* for dose to the organs at risk, the free breathing plans were used as the reference in the comparison (static plan). N2* for dose to the organs at risk, the convoluted free breathing plans were used as the reference in the comparison (convolved plan).

**Figure 3:**
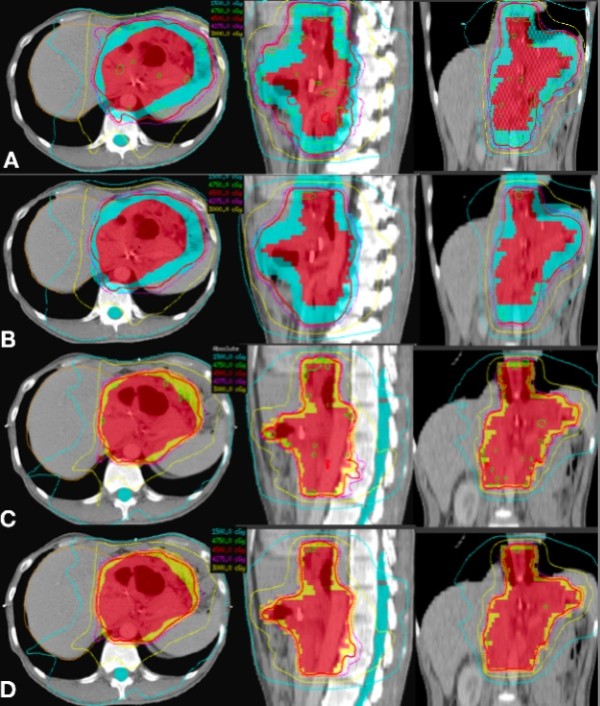
The dose distributions of one patient in free breath mode without (A) and with (B) convolving setup error and target motion and in combining ABC and online IGRT without (C) and with (D) convolving residual target motion.

All plans were similar in the target coverage with no significant statistic difference. The p values were 0.96 (for PTV V_45_, Vx means the percent volume that received x Gy) and 0.76 (for PTV V_42.75_) for static plans, and 0.78 (for V_45_) and 0.33 (for PTV V_42.75_) for convoluted plans. As for the critical structures, the IMRT_FB_ was chose as the reference in the comparison of liver V_30_ and kidneys V_15_. Static IMRT_IGBH_ plans had lower dose to the liver compared to static IMRT_FB_ (p = 0.01), but no significant difference was found in the kidney dose. However, after convolution, IMRT_IGBH_ showed better kidneys sparing (p < 0.01) but similar dose to the liver (p = 0.08). The dose distributions in the transverse, sagittal and coronal planes of one patient in the two scenarios are shown in Figure 3.

### The possibility of dose escalation

IMRT_IGBH_ plans with 54 Gy target dose were generated and compared with the original IMRT_FB_. As shown in Table3, both plans are convolved with the relative pdfs stated previously. The results show comparable target coverage and similar doses to liver, left kidney and spinal cord. The V_15_ of the right-kidney was smaller in IMRT_IGBH_.

**Table 3:** **Comparison of the dose to target and organs at risk for free breathing and breath-hold treatment techniques: the prescribed dose to the IMRT**_**FB**_**and IMRT**_**IGBH**_**were 45 Gy and 54 Gy, respectively**

	**PTV**_**FB**_**/PTV**_**IGBH**_		**Liver**	**Left kidney**	**Right kidney**	**Spinal cord**
	V_45_	V_42.75_	V_30_	V_15_	V_15_	Max Dose (Gy)
	Mean ± SD	Mean ± SD	Mean ± SD	Mean ± SD	Mean ± SD	
IMRT_FB_ (45 Gy)	95.5 ± 1.4%	98.8 ± 1.1%	30.3 ± 10.0%	47.5 ± 5.4%	40.2 ± 7.2%	41.6 ± 3.6%
IMRT_IGBH_(54 Gy)	96.0 ± 0.7%	98.8 ± 0.8%	31.0 ± 8.7%	47.2 ± 7.6%	34.1 ± 16.3%	43.4 ± 1.0%

## Discussion

In this study, we investigated the dosimetric benefit of combining image guidance and breath-hold technique for gastric cancer radiotherapy. We applied the convolution method in calculation of the actual delivered dose. And based on the results we also showed the feasibility of dose escalation from 45 Gy to 54 Gy with the image-guided breath-hold technique while keeping the dose to the organs at risk at the same level.

Compared to AP-PA, multiple-field 3DCRT and IMRT techniques offer better normal tissue sparing. Henning GT et al. [13]. reported an improved outcome associated with four or more radiation fields. Soyfer et al. [14]. reported that the complex 3DCRT techniques improved PTV coverage and lowered the doses to the kidneys and spinal cord. Therefore, the IMRT was used in this study to achieve conformal dose distribution to the target.

Setup errors and tumor motions are the two main factors in designing CTV to PTV margins for gastric cancer radiotherapy. For setup errors, we found that the mean and standard deviation was 0.0 ± 1.8 mm in LR, 1.2 ± 3.4 mm in SI and −1.4 ± 3.1 mm in AP direction, which were considered to follow the Gaussian distribution. Although our data was derived from the fluoroscopic imaging on the simulator, it represented the patient setup error because the same therapists positioned the patients on both the simulator and the actual treatment machines. However, the setup accuracy can be greatly improved with the online correction strategy [15]. Therefore, a 3 mm residual setup error after online image guided was applied for our study.

The target motion presents a challenge in radiotherapy treatment delivery. Motion degrades the reproducibility of the patient anatomic structures between the daily treatment and the initial CT acquisition, and can cause the dose to the tumor and normal organs differ from the original treatment plan. Our results showed a large margin was required for compensating the setup uncertainties and target motion in free breathing treatment. Large margins hinder the normal tissue sparing and target dose escalation.

The ABC technique has been widely used to reduce the respiratory induced organ motion. Wong et al. reported that the intra-fraction reproducibility of ABC was about 3 mm [8]. However, ABC can not eliminate the residual organ motion as well as the intra- and inter-fraction reproducibility uncertainties [16]. In our study, we evaluated the target motion within one ABC breath hold and the overall target motion between different breath-holds because each treatment fraction required several breath hold. We found the residual target motion were 3.7 mm, 1.6 mm and 2.8 mm in the SI, LR and AP directions, respectively. It is evident that it is possibility to reduce the margin in gastric cancer treatment using the breath hold technique.

Combining the daily image guidance and the breath hold technique can reduce the margin to 5–10 mm. Comparison of the static IMRT_FB_ and IMRT_IGBH_ plans demonstrated that the liver received lower dose in IMRT_IGBH_ (p = 0.01) while the target coverage remained the same (p > 0.05). Although no significant difference was found in kidneys (p = 0.09), the doses in IMRT_IGBH_ were slightly lower in some cases. The reason was that the margin in the LR direction for both FB and IGBH was about the same with a range of 5 to 7 mm. And compared to AP and SI directions, the margin in the LR direction had the prominent effect in the dose to the kidneys because of the anatomy positions of the kidneys. The margin in the AP direction determines how much the PTV overlaps with the liver, therefore lower dose is found in the IMRT_IGBH_.

Organ motion may lead to erroneous prediction of the actual delivered dose to the patient if the treatment plan is designed based on the static planning CT images. Although there are some limitations, the convolution method has been proposed to evaluate the changes in dose distributions during the treatment [17]. We incorporated the setup error and the free breathing target motion in IMRT_FB_. For the breath-hold plans, we assumed that the residual error from the online correction follow the Gaussian distributions. We found that both FB and IGBH had adequate target coverage. However, as for the liver and kidneys, doses were similar to or lower than the static plans (p < 0.01 for kidneys and p = 0.08 for liver), which imply that the actual kidneys (liver) sparing were better than (similar to) the planned one.

The ultimate goal of our investigation was to evaluate the possible dose escalation with the image guidance and ABC. Our study suggested that a significant dosimetric benefit could be achieved by combining the IMRT, image-guidance and breath-hold techniques in the treatment delivery. As the result, it is possible to escalate the target dose from 45 Gy to 54 Gy while keeping the critical organs under their dose tolerance. In other word, a better OAR sparing will be obtained if using the IGRT and ABC in a 45 Gy prescription. This will be a potential benefit to those patients with kidneys disease. This study is purpose on the dosimetric benefit on combining the IGRT and ABC techniques in the gastric cancer. Although the dose escalation in gastric radiotherapy is needed further verification, it is beyond the purpose of this dosimetric study.

## Conclusion

The gastric cancer radiotherapy requires a large margin to account for the setup error and organ motion in the free breathing treatment. The ABC technique can be used to reduce the gastric target motion, and further dosimetric benefit can be achieved by combining breath-hold and IGRT techniques in the adjuvant gastric cancer radiotherapy. It is feasible to escalate the target dose if the online correction strategy is implemented along with the breath-hold technique. The clinical implication and outcome needs further study.

## Competing interests

The authors declare that they have no competing interest.

## Authors’ contributions

Each author has participated sufficiently in the work to take public responsibility for appropriate portions of the content. ZZ and JY designed the study. WH, JW performed the study and data analysis. WH wrote the manuscript. QX performed the patient setup simulation. All authors read and approved the final manuscript.
